# Long term risk of distant metastasis in women with non‐metastatic breast cancer and survival after metastasis detection: a population‐based linked health records study

**DOI:** 10.5694/mja2.51687

**Published:** 2022-08-20

**Authors:** Sarah (Sally) J Lord, Benjamin Daniels, Belinda E Kiely, Dianne L O'Connell, Jane Beith, Sallie Pearson, Kim‐Lin Chiew, Max K Bulsara, Nehmat Houssami

**Affiliations:** ^1^ The University of Notre Dame Sydney NSW; ^2^ NHMRC Clinical Trials Centre the University of Sydney Sydney NSW; ^3^ Centre for Big Data Research in Health University of New South Wales Sydney NSW; ^4^ NHMRC Centre of Research Excellence in Medicines Intelligence Sydney NSW; ^5^ The Daffodil Centre the University of Sydney and Cancer Council NSW Sydney NSW; ^6^ The University of Newcastle Newcastle NSW; ^7^ Central Clinical School Chris O'Brien Lifehouse Sydney NSW; ^8^ Princess Alexandra Hospital Brisbane QLD; ^9^ Institute of Health Research University of Notre Dame Fremantle WA; ^10^ Sydney School of Public Health the University of Sydney Sydney NSW

**Keywords:** Breast neoplasms, Epidemiology

## Abstract

**Objectives:**

To estimate the long term risk of distant metastases (DM) for women with initial diagnoses of non‐metastatic breast cancer; to estimate breast cancer‐specific and overall survival for women with DM.

**Design:**

Population‐based health record linkage study.

**Setting, participants:**

Women diagnosed with localised or regional primary breast cancer recorded in the NSW Cancer Registry, 2001–2002.

**Major outcome measures:**

Time from breast cancer diagnosis to first DM, time from first DM to death from breast cancer. Secondary outcome: time to death from any cause.

**Results:**

6338 women were diagnosed with non‐metastatic breast cancer (localised, 3885; regional, 2453; median age, 59 years [IQR, 49–69 years]). DM were recorded (to 30 September 2016) for 1432 women (23%; median age, 62 years [IQR, 51–73 years]). The 14‐year cumulative DM incidence was 22.2% (95% CI, 21.1–23.2%; localised disease: 14.3% [95% CI, 13.2–15.4%]; regional disease: 34.7% [95% CI, 32.8–36.6%]). Annual hazard of DM was highest during the second year after breast cancer diagnosis (localised disease: 2.8%; 95% CI, 2.3–3.3%; regional disease: 9.1%; 95% CI, 7.8–10.3%); from year five it was about 1% for those with localised disease, from year seven about 2% for women with regional disease at diagnosis. Five years after diagnosis, the 5‐year conditional probability of DM was 4.4% (95% CI, 3.7–5.1%) for women with localised and 10.4% (95% CI, 9.1–12.0%) for those with regional disease at diagnosis. Median breast cancer‐specific survival from first DM record date was 28 months (95% CI, 25–31 months); the annual hazard of breast cancer death after the first DM record declined from 36% (95% CI, 33–40%) during the first year to 14% (95% CI, 11–18%) during the fourth year since detection.

**Conclusions:**

DM risk declines with time from diagnosis of non‐metastatic breast cancer, and the annual risk of dying from breast cancer declines with time from initial DM detection. These findings can be used to inform patients at follow‐up about changes in risk over time since diagnosis and for planning health services.

**The known:** Survival for women with breast cancer is improving, but population estimates of long term distant metastasis risk and survival with metastases are limited.

**The new:** In Australia, the annual risk of distant metastases declines with time from breast cancer diagnosis, to about 1% five years (localised cancer) or 2% seven years (regional cancer) after diagnosis. The annual risk of dying from breast cancer is 36% during the first year and 14% during the fourth year after metastasis detection.

**The implications:** Our findings can inform discussions with patients about the expected risks of distant metastases and subsequent death from breast cancer, as well as treatment and support service planning.

Breast cancer is the second most frequently diagnosed cancer in Australia (estimated 20 030 new diagnoses in 2021, second only to prostate cancer).^1^ At the time of diagnosis, 95% of women have no distant metastases (DM) and their long term survival is high.^2^ However, DM can develop later, even years after completing primary treatment.^3^

Accurate information about the likelihood of DM after early stage disease is important for preparing patients without causing them undue worry.^4^ Based on an analysis of New South Wales Cancer Registry and hospitals data, we previously estimated that the population‐level risk of DM five years after diagnosis of breast cancer was 10%.^5^ More recently, another group analysed Pharmaceutical Benefits Scheme (PBS) and Medicare Benefits Schedule (MBS) treatment claims data to more accurately estimate the date of first DM. For women who had not experienced recurrence by 18 months, the authors found that the risk of loco‐regional or distant recurrence was 3.3% per year during the six years following diagnosis of non‐metastatic breast cancer.^6^

As most women are DM‐free five years after diagnosis, estimates of longer term risk are needed. Meta‐analyses of adjuvant therapy trials have provided estimates for breast cancer subtypes, including oestrogen receptor (ER)‐positive tumours at twenty years^3^ and *HER2*‐positive tumours at ten years.^7^ However, population estimates are essential for understanding the prognosis for all women with breast cancer.

Risk factor information, such as cancer stage and receptor status,^8^ informs prognostic discussions at diagnosis. However, DM risk changes with time, and is greatest during the years immediately following diagnosis.^5^ Information that takes into account the time a person has remained DM‐free is accordingly most relevant for prognostic discussions during follow‐up after primary breast cancer treatment. People who develop DM also need information about expected survival.^9^ Population‐based cancer registries do not routinely report on progression to DM or subsequent survival. Cancer registry 5‐year survival estimates for people with *de novo* metastatic breast cancer^2^ (a minority of those with DM) may not apply to people who develop metastases after diagnosis.^10^

We therefore sought to estimate the 14‐year risk of DM for women with an initial diagnosis of non‐metastatic breast cancer, as well as the change in annual risk with time after breast cancer diagnosis; breast cancer‐specific survival and overall survival for women with DM; and changes in the annual risk of dying from breast cancer after DM detection.

## Methods

We conducted a population‐based retrospective cohort study based on health record linkage of routinely collected health data. We included data for women aged 18 years or more diagnosed with invasive primary breast cancer first registered in the NSW Cancer Registry during 1 January 2001 – 31 December 2002 as localised (confined to breast tissue, no axillary lymph node spread [T1 to T3 N0]) or regional breast cancer (node‐positive, or locally advanced [T4]). We excluded men, and women with DM or unknown disease extent at diagnosis, no linked NSW hospital records, or non‐breast primary cancer prior to their breast cancer diagnosis, and also those who died within 30 days of breast cancer diagnosis.

### Data sources

Our data sources were the NSW Cancer Registry, NSW Registry of Births, Deaths and Marriages (RBDM), Cause of Death Unit Record Files (COD‐URF), the NSW Admitted Patient Data Collection (APDC), and PBS and MBS claims data (Supporting Information, section 1). We have reported details of these datasets and their linkage in the protocol for our broader research program.^11^

We extracted data on age, remoteness of residence (major city, inner regional, outer regional, and remote/very remote, according to the Accessibility and Remoteness Index of Australia, ARIA),^12^ postcode‐level socio‐economic status (Index of Relative Socio‐economic Disadvantage quintile),^13^ country of birth (Australia or New Zealand, other), extent of disease (NSW Cancer Registry classification: localised or regional), and tumour morphology (by International Classification of Diseases for Oncology, version 3 [ICD‐O‐3] morphology code: ductal, lobular or mixed ductal/lobular, other). We deemed tumour ER status to be positive if adjuvant endocrine treatment had been provided. To avoid survivor bias, ER assessment was based on prior treatment for people who had been DM‐free for at least two years (for analysis of DM risk) or at the time of first DM record (for analysis of survival after DM detection).

### Outcomes

Primary outcomes were time from breast cancer diagnosis to first DM, and from first DM to death from breast cancer; time from first DM to death from any cause was the secondary outcome. We estimated the date of first DM from each dataset to 30 September 2016, the final date for which NSW Cancer Registry metastatic disease notifications data were available (Supporting Information, section 1). We determined the date and cause of death from RBDM and COD‐URF data to 30 June 2017.

### Statistical analysis

Time from primary breast cancer diagnosis (NSW Cancer Registry) to first DM was derived from the date of the earliest record meeting our DM criteria or of DM recorded at death (Supporting Information, section 1). We calculated the cumulative incidence function to estimate the DM risk to 14 years (with 95% confidence intervals, CIs) overall and by extent of disease, age (under 40, 40–49, 50–69, 70 years or more), tumour morphology (ductal, lobular/mixed, other), remoteness of residence, and socio‐economic status. A DM record later than a NSW Cancer Registry record of a non‐breast primary cancer and non‐breast cancer death were treated as competing events.^14^ We censored data for participants without DM records or competing events at 30 September 2016. We estimated the conditional probability of DM within five years after each year of accumulated DM‐free time (1–9 years) by repeating the analysis for women without DM at the beginning of each conditional time point.

We report changes in DM risk with time since breast cancer diagnosis in plots of the annual DM hazard rate, estimated at the midpoint of each year, by extent of disease at diagnosis, age group, tumour morphology, and tumour ER status. To provide context, we also report graphically the annual hazards for breast cancer and non‐breast cancer death.

We report initial DM sites using the International Classification of Diseases, tenth revision, Australian modification (ICD‐10‐AM) diagnosis codes (as recorded in the APDC) for secondary malignant neoplasms, and categorise the number of visceral sites as zero, one, or two or more. We estimated breast cancer‐specific survival from the date of the first DM record to the date of breast cancer death. We used the Kaplan–Meier method to estimate median breast cancer‐specific and overall survival, both overall and by subgroup, age at first DM record, and time to first DM (less than two, 2–5, more than five years). We censored data at the date of non‐breast cancer death or 30 June 2017 to estimate median breast cancer‐specific survival. We treated non‐breast cancer deaths as competing events to estimate the 5‐year incidence of breast cancer death after first DM. We report changes in the annual risk of breast cancer death over time since first DM record in plots of the annual hazard rate for breast cancer death. Statistical analyses were conducted in SAS 9.4.

### Ethics approval

The research ethics committees of the Australian Institute of Health and Welfare (EO2017/2/255), NSW Population and Health Services (HREC/17/CIPHS/19), and the University of Notre Dame Australia (0‐17‐144S) approved our study.

## Results

We analysed data for 6338 women diagnosed with non‐metastatic breast cancer during 2001–2002 (localised, 3885; regional, 2453) (Supporting Information, figure 2); their median age was 59 years (interquartile range [IQR], 49–69 years) (Box 1). Subsequent DM records (to 30 September 2016) were identified for 1432 women (23%), including 557 (39%) more than five years after breast cancer diagnosis; the median age at first DM record was 62 years (IQR, 51–73 years). Of the 1119 people for whom initial DM sites was recorded (78%), the most frequent were bone (524, 47%), lung or pleura (375, 34%), and liver (331, 30%) (Box 2).

Box 1 **Characteristics of women diagnosed with non‐metastatic breast cancer, New South Wales, 2001–2002, and of women without distant metastases five and ten years after breast cancer diagnosis**

**Table:** 

	Women without distant metastases		
Characteristic	At diagnosis	At five years	At ten years
Women	6338	5137	4406
Extent of disease			
Localised (T1–T3 N0)	3885 (61%)	3372 (66%)	2949 (67%)
Regional (T4 or N+)	2453 (39%)	1765 (34%)	1457 (33%)
Age (years)			
Median (interquartile range)	59 (49–69)	64 (55–74)	68 (59–77)
Under 40	386 (6%)	82 (2%)	22 (1%)
40–49	1223 (19%)	637 (12%)	225 (5%)
50–59	1725 (27%)	1293 (25%)	918 (21%)
60–69	1423 (22%)	1419 (28%)	1325 (30%)
70 or more	1581 (25%)	1706 (33%)	1916 (43%)
Tumour morphology			
Invasive ductal	4874 (77%)	3933 (77%)	3387 (77%)
Invasive lobular/mixed	806 (13%)	668 (13%)	553 (13%)
Other	658 (10%)	536 (10%)	466 (11%)
Treatment‐defined estrogen receptor status (prior endocrine therapy)*	—	3451 (70%)	2808 (67%)
Any endocrine or chemotherapy records	5190 (82%)	4117 (80%)	3475 (79%)
Remoteness of residence			
Major city	4555 (72%)	3688 (72%)	3175 (72%)
Inner regional	1353 (21%)	1099 (21%)	923 (21%)
Outer regional	400 (6%)	323 (6%)	285 (6%)
Remote/very remote	30 (0.5%)	27 (0.5%)	23 (1%)
Postcode‐level socio‐economic status			
1 (most disadvantaged)	1056 (17%)	836 (16%)	718 (16%)
2	1266 (20%)	1003 (20%)	817 (19%)
3	1213 (19%)	987 (19%)	853 (19%)
4	1182 (19%)	964 (19%)	841 (19%)
5 (least disadvantaged)	1621 (26%)	1347 (26%)	1177 (27%)
Country of birth			
Australia/New Zealand	4330 (71%)	3451 (70%)	2931 (70%)
Other	1769 (29%)	1469 (30%)	1263 (30%)
Missing data	239	217	212
Subsequent non‐breast primary cancer during follow‐up	525 (8%)	397 (8%)	255 (6%)

* Based on number of women for whom endocrine therapy was dispensed in the preceding period, expressed as a proportion of the number with linked Pharmaceutical Benefits Scheme records (6151 women).

Box 2 **Characteristics of women diagnosed with non‐metastatic breast cancer, New South Wales, 2001–2002, with distant metastasis record (to 30 September 2016), and for women alive five years after the first distant metastasis record**

**Table:** 

Characteristic	Women with distant metastasis	Women alive five years after distant metastasis detected
Women	1432	333
Extent of disease (at diagnosis)		
Localised (T1–T3 N0)	570 (40%)	161 (48%)
Regional (T4 or N+)	862 (60%)	172 (52%)
Age (years)		
Median (interquartile range)	62 (51–73)	62 (54–71)
18–49	312 (22%)	43 (13%)
50–69	677 (47%)	195 (59%)
70 or more	443 (31%)	95 (29%)
Tumour morphology		
Invasive ductal	1125 (78%)	259 (78%)
Invasive lobular/mixed	196 (14%)	51 (15%)
Other	111 (8%)	23 (7%)
Time to distant metastasis (years)		
Less than two	472 (33%)	143 (43%)
2–5	403 (28%)	88 (26%)
More than five	557 (39%)	102 (31%)
Visceral sites (first record): location (multiple sites possible)		
Bone	524 (47%)	59 (40%)
Lung/pleura	375 (34%)	33 (22%)
Liver	331 (30%)	23 (16%)
Brain	139 (12%)	10 (7%)
Lymph/other	174 (16%)	47 (32%)
Not recorded	313	185
Visceral sites (first record): number		
0	449 (40%)	94 (64%)
1	517 (46%)	44 (30%)
2 or more	153 (14%)	10 (7%)
Prior adjuvant systemic therapy*		
Endocrine	949 (67%)	250 (76%)
Cytotoxic	525 (37%)	106 (32%)
Remoteness of residence		
Major city	1034 (72%)	248 (74%)
Inner regional	309 (22%)	72 (22%)
Outer regional/remote/very remote	89 (6%)	13 (4%)
Postcode‐level socio‐economic status		
1 (most disadvantaged)	259 (18%)	51 (15%)
2	322 (22%)	80 (24%)
3	254 (18%)	48 (14%)
4	251 (18%)	71 (21%)
5 (least disadvantaged)	346 (24%)	83 (25%)
Country of birth		
Australia/New Zealand	1012 (72%)	237 (73%)
Other	398 (28%)	87 (27%)
Not recorded	22	9

* Expressed as proportion of the number with linked Pharmaceutical Benefits Scheme records (1419 women).

### Cumulative incidence and annual hazard of distant metastasis

The 14‐year cumulative DM incidence was 22.2% (95% CI, 21.1–23.2%); for women diagnosed with localised breast cancer it was 14.3% (95% CI, 13.2–15.4%), for those with regional breast cancer 34.7% (95% CI, 32.8–36.6%). In both cases, the incidence among women diagnosed before 40 years of age was higher than for older women (Box 3). The 14‐year DM risk was not influenced by residential remoteness, but was higher for women in the two most socio‐economically disadvantaged quintiles than for those in the less disadvantaged quintiles (Box 4).

Box 3 **Cumulative incidence of distant metastasis, by extent of disease and age at breast cancer diagnosis**

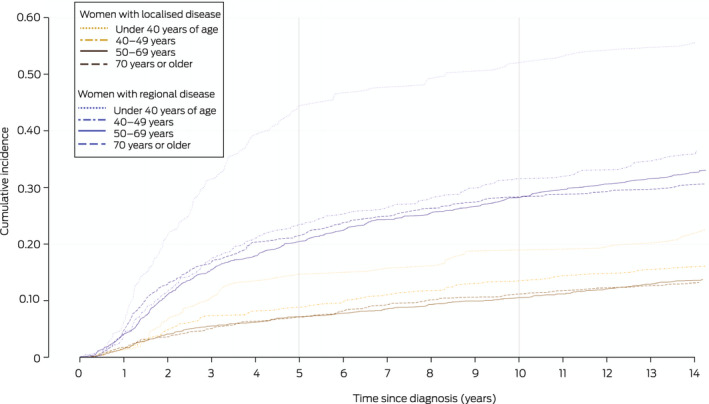

Box 4 **Cumulative incidence of distant metastasis in women diagnosed with non‐metastatic breast cancer, New South Wales, 2001–2002**

**Table:** 

	Cumulative incidence of distant metastasis (95% CI)			
Characteristic	5‐year	10‐year	14‐year	*P* *
All women	13.8% (12.9–14.6%)	19.1% (18.1–20.0%)	22.2% (21.1–23.2%)	
**Women with localised cancer**	7.8% (7.0–8.7%)	11.6% (10.6–12.7%)	14.3% (13.2–15.4%)	
Age (years)				0.006
Under 40	14.2% (9.8–19.5%)	18.8% (13.7–24.5%)	21.9% (16.4–28.0%)	
40–49	8.8% (6.8–11.2%)	13.5% (10.9–16.3%)	15.9% (13.1–18.9%)	
50–69	7.2% (6.1–8.4%)	10.6% (9.3–12.0%)	13.6% (12.1–15.1%)	
70 or more	7.1% (5.7–8.8%)	11.2% (9.4–13.2%)	13.1% (11.2–15.3%)	
**Women with regional cancer**	23.2% (21.6–24.9%)	30.9% (29.0–32.7%)	34.7% (32.8–36.6%)	
Age (years)				< 0.001
Under 40	44.4% (37.2–51.4%)	51.9% (44.5–58.7%)	55.0% (47.6–61.8%)	
40–49	23.5% (20.1–26.9%)	31.5% (27.8–35.3%)	35.7% (31.9–39.6%)	
50–69	20.4% (18.1–22.8%)	28.2% (25.6–30.8%)	32.6% (29.9–35.4%)	
70 or more	21.5% (18.0–25.3%)	28.3% (24.4–32.3%)	30.4% (26.4–34.5%)	
**Demographic characteristics**				
Tumour morphology				0.002
Invasive ductal	14.5% (13.5–15.5%)	19.7% (18.6–20.9%)	22.7% (21.5–23.9%)	
Invasive lobular/mixed	11.8% (9.7–14.2%)	18.9% (16.2–21.6%)	23.4% (20.5–26.4%)	
Other	10.7% (8.5–13.2%)	14.4% (11.8–17.2%)	16.7% (14.0–19.7%)	
Remoteness of residence				0.63
Major city	13.9% (12.9–14.9%)	19.2% (18.0–20.3%)	22.3% (21.1–23.5%)	
Inner regional	13.7% (11.9–15.6%)	19.2% (17.1–21.4%)	22.4% (20.2–24.7%)	
Outer regional/remote/very remote	12.6% (9.7–15.9%)	17.5% (14.1–21.2%)	20.5% (16.8–24.5%)	
Postcode‐level socio‐economic status				0.010
1 (most disadvantaged)	15.8% (13.6–18.0%)	20.4% (18.1–22.9%)	24.2% (21.6–26.8%)	
2	15.8% (13.8–17.8%)	22.3% (20.0–24.6%)	24.9% (22.6–27.4%)	
3	12.6% (10.8–14.5%)	17.6% (15.5–19.8%)	20.7% (18.5–23.0%)	
4	13.4% (11.6–15.4%)	18.1% (16.0–20.4%)	20.6% (18.4–23.0%)	
5 (least disadvantaged)	12.0% (10.5–13.6%)	17.4% (15.6–19.3%)	20.8% (18.8–22.8%)	

* For 14‐years; Gray’s test for equality of cumulative incidence.^15^

The annual hazard rate for first DM (ie, 12‐month probability for women alive and DM‐free at a given time point) was highest during the second year after breast cancer diagnosis, both for women with localised (2.8%; 95% CI, 2.3–3.3%) and those with regional cancer (9.1%; 95% CI, 7.8–10.3%). The annual hazard was fairly stable from year five for those with localised disease (about 1%) and from year seven for women with regional disease (about 2%) (Box 5; Supporting Information, table 2).

Box 5 **Annual hazards of first distant metastasis, breast cancer death, and other cause death, by time since diagnosis of non‐metastatic breast cancer, extent of initial disease, and age group**

**Figure:**
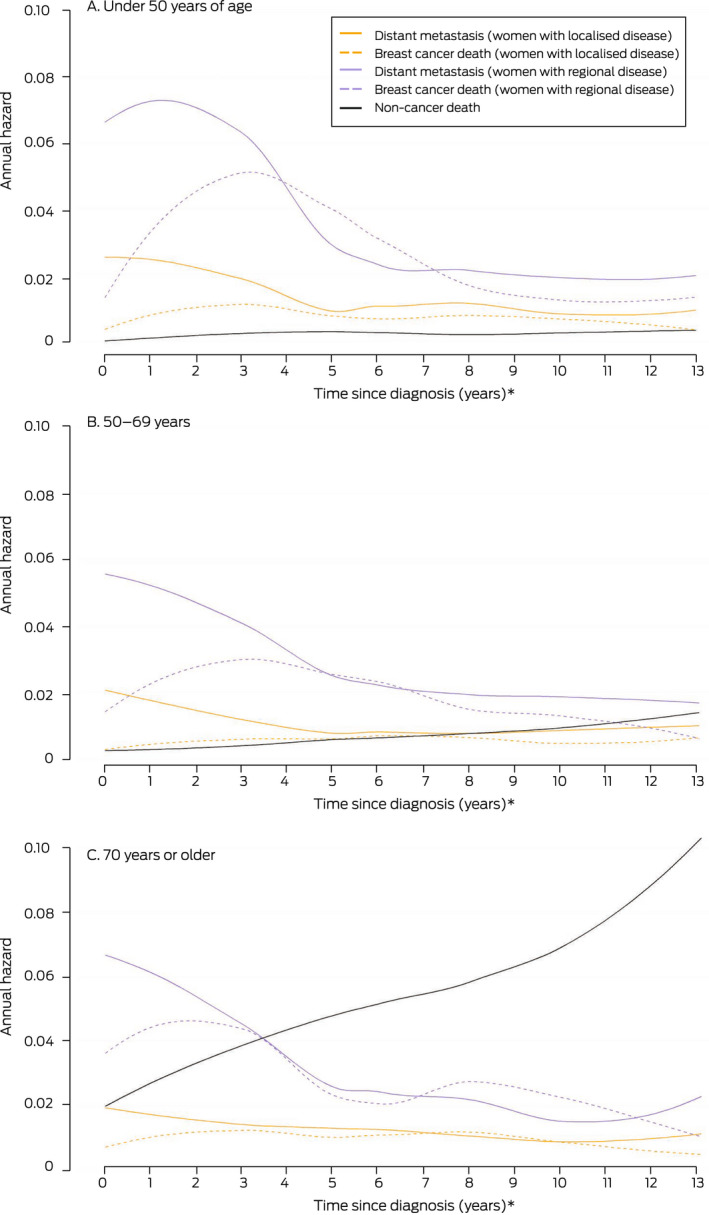
* At each time point, the graph depicts the risk of the specified event over the next twelve months. This is distinct from cumulative incidence estimates, which express the risk of DM to a designated time point.

The 5‐year conditional probability of DM declined with time from diagnosis for women with localised or regional disease, but was higher for those with regional disease at all time points. Five years after diagnosis, the conditional probability of DM during the following five years was 4.4% (95% CI, 3.7–5.1%) for women with localised and 10.4% (95% CI, 9.1–12.0%) for those with regional disease at diagnosis (Box 6).

Box 6 Five‐year conditional probability of distant metastasis, by time since breast cancer diagnosis and disease extent at diagnosis*

**Figure:**
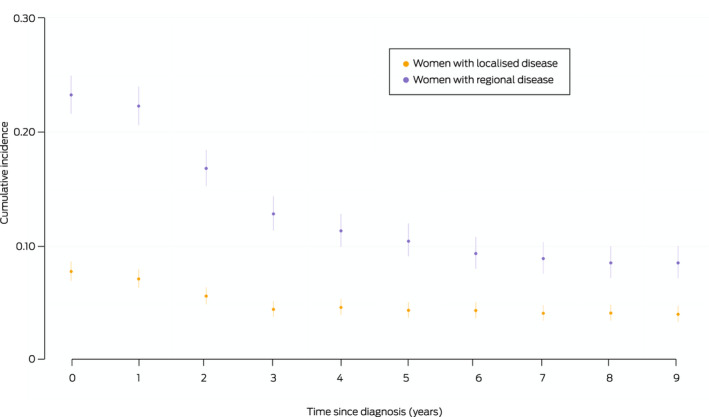
* Each point indicates the probability of a first distant metastasis being recorded during the subsequent five years, with its 95% confidence interval.

For both women with localised and regional disease, the annual hazard of DM was initially higher for women under 50 years of age than for older women, for women with ductal than for those with lobular tumours, and for women with ER‐negative cancers, but the differences had largely dissipated five years after diagnosis (Supporting Information, figure 3).

### Overall and breast cancer‐specific survival

The annual hazard of breast cancer death peaked for women in all age groups with localised and regional disease at diagnosis two to three years after diagnosis. The annual hazard of death from other causes (localised and regional breast cancer groups combined) increased with time; for women aged 50–69 years, it was greater than the annual hazards of DM and breast cancer death seven years after diagnosis of localised breast cancer, and for women aged 70 years or more four years after diagnosis of regional disease (Box 5).

Median breast cancer‐specific survival from first DM record date was 28 months (95% CI, 25–31 months); median overall survival was 23 months (95% CI, 21–26 months), and 5‐year overall survival was 29% (95% CI, 27–32%). Median breast cancer‐specific and overall survival were influenced by age, disease extent at breast cancer diagnosis, time to first DM, number of metastasis sites, remoteness of residence, and treatment‐defined ER status (Box 7).

Box 7 **Overall and breast cancer‐specific survival after first distant metastasis record**

**Table:** 

Characteristic	Number	All‐cause deaths	Overall survival (months), median (95% CI)	IQR	*P* *	Breast cancer deaths	Breast cancer‐specific survival (months), median (95% CI)	IQR	*P* *
All women	1425^†^	1049	23 (21–26)	6–88		900	28 (25–31)	8–NR	
Age (years) (first distant metastasis)					< 0.001				< 0.001
18–49	310	236	27 (23–31)	9–98		228	27 (23–32)	10–111	
50–69	675	461	30 (26–34)	9–NR		413	34 (30–38)	11–NR	
70 or more	440	352	13 (9–16)	2–50		259	20 (16–24)	4–119	
Disease extent at diagnosis					< 0.001				< 0.001
Localised	566	376	31 (25–36)	8–NR		289	42 (35–56)	12–NR	
Regional	859	673	20 (18–23)	5–59		611	22 (20–25)	6–72	
Tumour morphology					0.11				0.13
Ductal	1118	825	23 (20–25)	6–90		713	27 (24–31)	8–34	
Lobular/mixed	196	138	30 (22–38)	9–95		115	35 (27–48)	13–39	
Other	111	86	15 (9–23)	2–60		72	20 (13–34)	5–30	
Remoteness of residence					0.13				0.043
Major city	1030	763	23 (20–26)	6–87		647	29 (25–34)	8–NR	
Inner regional	306	216	23 (20–30)	6–145		188	28 (22–33)	8–NR	
Outer regional/remote	89	70	16 (9–27)	3–42		65	18 (13–27)	6–44	
Socio‐economic status					0.08				0.09
1–3 (more disadvantaged)	829	624	22 (19–25)			537	26 (23–30)	7–NR	
4–5 (less disadvantaged)	596	425	24 (20–30)			363	32 (26–40)	9–NR	
Time to distant metastasis (years)					< 0.001				< 0.001
Less than two	465	354	18 (15–21)	5–148		310	20 (16–26)	5–NR	
2–5	403	345	20 (18–24)	5–51		310	23 (19–26)	7–59	
More than five	557	350	31 (27–36)	9–103		280	40 (34–49)	15–NR	
Visceral sites (first hospital record)^‡^					< 0.001				< 0.001
0	449	353	26 (22–29)	11–62		305	29 (26–34)	13–77	
1	511	469	11 (9–14)	3–31		414	15 (12–18)	4–35	
2 or more	153	150	9 (5–12)	3–22		141	9 (6–14)	4–24	
ER status^§^					< 0.001				< 0.001
ER‐positive	946	652	30 (26–34)	9–157		548	36 (32–42)	12–NR	
ER‐negative	420	344	16 (13–18)	5–45		318	18 (15–20)	5–53	

ER = estrogen receptor; IQR = interquartile range; NR = not reached (ie, beyond censoring point).

* Log rank test for differences in the survival distribution.

† Excludes women with distant metastases recorded at death only.

‡ Excludes 312 women with no hospital records of distant metastasis site.

§ ER‐positive: endocrine therapy prior to first distant metastasis record; ER‐negative: cytotoxic therapy only prior to first distant metastasis.

The annual hazard of breast cancer death after the first DM record declined from 36% (95% CI, 33–40%) during the first year after detection to 14% (95% CI, 11–18%) during the fourth year (Supporting Information, table 3). Similar declines were noted in subgroup analyses, except for women with two or more visceral metastases; differences by age group, time to first DM, and tumour ER status also declined with time from first DM (Supporting Information, figure 4).

Sensitivity analyses applying less or more stringent definitions of first DM yielded results similar to our main analyses for both the cumulative incidence of DM and breast cancer‐specific survival (Supporting Information, table 4).

## Discussion

We estimate that 22.2% of Australian women diagnosed with non‐metastatic breast cancer during 2001–2002 developed DM within 14 years. For women initially diagnosed with localised breast cancer, the risk of DM within the next ten years was 11.6% (about 1 in 9), and 4.4% (1 in 23) within the next five years for those DM‐free at five years; for women with regional breast cancer, the 10‐year risk at diagnosis was 30.9% (about 1 in 3) and the 5‐year risk five years after diagnosis 10.4% (1 in 10).

Survival time from the first DM record varied widely. Overall median survival was 23 months (IQR, 6–88 months), but 29% of women were alive five years after DM detection. Prognosis was poorest for those aged 70 years or more at the time of their first DM record (31% of women with DM). That the annual risk of breast cancer death declined with time from the first DM record is probably explained by the heterogeneity of metastatic breast cancer: some people have aggressive disease that leads to death within two years, others have more indolent, treatment‐responsive disease and live longer than five years. Our findings illustrate the importance of adjusting prognostic information according to the number of event‐free years and accordingly revising discussions with patients over time.

The DM risk for women more recently diagnosed with breast cancer is probably lower than we found. More effective adjuvant therapies have been listed by the PBS since 2002, including aromatase inhibitors (2004)^16^ and trastuzumab (2006).^17^ A population‐based New Zealand study estimated that 10‐year DM risk was 16.5% (95% CI, 15.8–17.2%) for non‐metastatic breast cancer diagnosed during 1991–2017,^18^ lower than our 10‐year estimate (19.1%; 95% CI, 18.1–20.0%). An Australian Capital Territory cohort study recorded 10‐year recurrence rates of 10.6% (luminal), 24.4% (*HER2*‐positive), and 26.4% (triple‐negative tumours) for breast cancer diagnosed during 2002–2015; DM accounted for 72% of recurrences.^19^ European cancer registry studies have reported 10‐year DM risks ranging from 6.0% (Germany, 1999–2009)^8^ and 7.8% (the Netherlands, 2005)^20^ for T1/2 N0 tumours to 27.3% for resected T3/4 node‐positive tumours.^8^ The Dutch study also reported declining risk over time since diagnosis,^20^ similar to our findings.

Our finding of 23 months median overall survival after DM detection is similar to that of a large Munich Cancer Registry study (1998–2013; 23 months)^21^ and longer than reported by a New Zealand study (2010–2017; 18 months).^22^ Our finding of substantially longer median overall survival for women with treatment‐defined ER‐positive tumours (30 months) than those with ER‐negative tumours (16 months) is consistent with those of overseas studies.^22^, ^23^

As treatment of DM with trastuzumab during 2001–2015 was not covered by the PBS, we could not separately identify *HER2*‐positive DM for our analysis. In the whole‐of‐population study of all 4177 Australian women with *HER2*‐positive metastatic breast cancer who commenced trastuzumab under the Herceptin Program during 2001–2011, median overall survival was 28.1 months (IQR, 12.6–62.9 months); 26% of the women were alive at five years.^24^ Our finding of longer median overall survival (31 months; 95% CI, 27–36 months) for women in whom DM were detected more than five years after breast cancer diagnosis (ie, during 2007–2016) may thus reflect both favourable tumour prognostic factors and the impact of more effective treatments for DM. Longer survival can be expected for women eligible for newer treatments, such as cyclin‐dependent kinase 4/6 inhibitors for ER‐positive, *HER2*‐negative metastatic breast cancer (MONALEESA‐2 trial: median overall survival, 62.3 months (95% CI, 52.4–71.0 months).^25^

Cancer registry initiatives in Australia^2^ and overseas^26^ for reporting 5‐year conditional survival for people with breast cancer recognise the importance of event‐free time. Our study helps in this respect by providing population‐level DM data. Although not designed to provide individualised prognostic information, our findings can inform discussions with women completing treatment for breast cancer about the DM risk at each year of follow‐up, and the risk of death from breast cancer after DM detection. Although absolute DM risk levels will be lower for women receiving newer adjuvant therapies, the reported changes in risk with time since diagnosis will remain informative. We provide graphic summaries for each age group to facilitate discussions, and include the risk of death from other causes to place risks into context. For ER‐positive cancer, DM risk for women with regional disease declined markedly over the first five years after diagnosis but remained higher than for those with localised disease. For ER‐negative cancer, DM risk approached that for ER‐positive disease by five years, consistent with findings regarding triple‐negative disease.^27^, ^28^ Our finding that DM risk in ER‐negative regional disease was similar to that for localised disease from nine years raises questions for further prognostic research.

### Limitations

Using administrative health records to identify the date of first DM, without comparison with clinical records, entails a risk of bias. Assuming all DM can be identified in these records at some point before death, the main direction would be toward longer time to DM, leading to underestimation of DM at early time points (eg, 5‐year DM risk) and subsequent survival. However, only 58 breast cancer deaths were not linked with prior DM records (Supporting Information, table 4), so the risk of missed ascertainment was probably low. Conversely, misclassification of records as DM events would lead to overestimating DM risk and subsequent survival. Further, our survival estimates by DM site excluded 312 women for whom this information was unavailable, and we had no information on disease stage at diagnosis, tumour grade, or tumour receptor subtype.

### Conclusion

Our findings for the long term risk of DM are important for planning health services, including treatment and support for people diagnosed with DM, as outlined in the 2019 Cancer Australia statement^9^ and the Victorian optimal care pathway for people with breast cancer.^29^ For health service planners, the higher DM risk for women living in socio‐economically disadvantaged areas and shorter survival with DM for those in outer regional or remote areas suggest health inequities that require attention. As it is important to monitor the impact of services and treatment advances on DM risk and survival, routine notification, validation, and reporting of DM across Australia is imperative, data routinely collected in the United Kingdom^30^ and New Zealand.^31^ In Australia, there is no legal requirement to report DM. However, increased use of electronic data collection systems for sending notifications to registries allows more complete data collection that will be essential for new initiatives.

## Open access

Open access publishing facilitated by The University of Notre Dame Australia, as part of the Wiley – The University of Notre Dame Australia agreement via the Council of Australian University Librarians.

## Competing interests

Belinda Kiely has received funding for advisory board participation (Roche, Gilead), meeting registration fees (Novartis), and an educational presentation (Novartis). Jane Beith has received funding for conference attendance (Novartis).

## Supporting information


Appendix S1.
