# Supplementary material for: Long term risk of distant metastasis in women with non‐metastatic breast cancer and survival after metastasis detection: a population‐based linked health records study
Source: Med J Aust. 2022 Aug 20;217(8):402–9. doi: 10.5694/mja2.51687 (PMC9804703; doi:10.5694/mja2.51687)
Supplement: Supplementary file 1 — Appendix S1. [file MJA2-217-402-s001.pdf]

MJA
The Medical Journal of Australia

Supporting Information

Supplementary methods and results

**This appendix was part of the submitted manuscript and has been peer reviewed.
It is posted as supplied by the authors.**

Appendix to: Lord SJ, Daniels B, Kiely BE, et al. Long term risk of distant metastasis in women with non-metastatic breast cancer and survival after metastasis detection: a population-based linked health records study. *Med J Aust* 2022; doi: 10.5694/mja2.51687.

1. Supplementary methods

Data sources, access, linkage

To create the study cohort, the New South Wales Cancer Registry (NSWCR) data custodian identified eligible people diagnosed with breast cancer and provided the Centre for Health Record Linkage (CHeReL) with a list of corresponding registry record identifiers. The CHeReL created a Project Person Number (PPN) for each person and performed the record linkage for the NSW-held datasets (NSWCR, Admitted Patient Data Collection (APDC), Registry of Births, Deaths and Marriages (RBDM), Cause of Death Unit Record File (COD-URF)). The Australian Institute of Health and Welfare (AIHW) performed the record linkage for the national datasets (Pharmaceutical Benefits Scheme (PBS), Medicare Benefits Schedule (MBS)). Each data custodian extracted the approved study variables for uploading into a secure project workspace within the Sax Institute Secure Unified Research Environment (SURE) facility. Investigators (SL, BD) used the SURE project workspace on a Virtual Desktop Infrastructure environment to access study data and perform analyses.

The study period commenced in 2001, the earliest year for which APDC records were available for linkage, to allow estimation of long term distant metastasis (DM) outcomes. We selected a two-year cohort to obtain precise estimates of the cumulative incidence of DM for population study subgroups.

We did not have access to BC screening participation for the study cohort. However, women living in NSW aged 40 years or more have had access to the NSW BreastScreen program since 1991. Participation in biennial BC screening for women aged 50-69 years (the target age group in 2001-2002) was reported as approximately 53% in 2000-2001 in NSW, with the same participation rate reported in 2018-2019 (the most recent monitoring report).[1,2]

Distant metastasis: definition and criteria

We defined DM as clinical, radiological or pathological evidence of metastasis in distant organs or non-regional lymph nodes (AJCC 8th edition).[3] We developed six criteria to estimate the date of the first DM from administrative health records of a diagnosis or treatment of DM. These criteria included: four 'metastatic-specific' criteria from cancer registry notifications, hospital admissions, PBS and MBS records that specify metastatic disease; and two 'metastatic pattern' criteria from PBS and MBS records for medicines and radiation oncology services that are not restricted for use in metastatic cancer but the timing of use is highly consistent with treatment for metastatic disease. We defined loco-regional recurrence and contralateral/second primary BC from any further hospital procedure codes for breast or axillary lymph node surgery, or MBS codes for radiotherapy to the breast after treatment of the primary cancer; or a cancer registry notification of a second primary BC. We did not include these events as DM (see table below). The NSW Cancer Registry receives notifications of new and recurrent cases of cancer as a statutory requirement for pathology laboratories, public and private hospitals, departments of radiation oncology, outpatient departments, day procedure centres and nursing homes. For our analysis of risk of DM, if a person had no

other record of DM but "secondary malignant neoplasm" was listed as a contributing cause of death together with no record of another (non-breast) cancer, we used the date of death as the date of first DM.

Table 1. Health record criteria for estimating date of first distant metastasis

Data source	Criteria
Metastatic-specific	
NSWCR	1. Cancer registry notification record of first distant metastasis.
APDC	2. Hospital diagnosis code for secondary malignant neoplasm (ICD 10-AM C77, C77.1, C77.2, C77.4-C77.8, C78.0–C78.8, C79.0–C79.88), excludes lymph nodes to axillary/upper limb and neck (to exclude supraclavicular nodes).
MBS	3. Radiation service that specifies 'secondary site'.<br>These metastatic-specific MBS items were introduced in May 2003.
PBS	4. Anti-neoplastic drug, use restricted to advanced or metastatic breast cancer.
Metastatic treatment pattern	
PBS	5. Anti-neoplastic drug, use not restricted to metastatic disease.<br>Treatment initiated after the initial adjuvant treatment period (defined as ≤12 months after the primary BC registration date in the NSWCR); and after a treatment gap ≥90 days from prior adjuvant therapy; and ≥90 days before or after health records indicating locoregional recurrence or a second primary BC (defined as a hospital record for breast or axillary lymph node surgery, an MBS item for radiation that specified site as primary BC, or a second primary BC record in the NSWCR).
MBS	6. Radiation service that does not distinguish between primary and secondary sites.<br>These non-specific MBS items were discontinued in April 2003.<br>Treatment initiated after the initial adjuvant treatment period. Palliation therapy was defined as <15 sequential (fractionated) services and accepted for this criterion. Adjuvant therapy was defined as ≥25 sequential fractionated services, corresponding to standard practice at the time, and was not accepted for this criterion. Radiation services between these limits were reviewed with other health records with radiation oncologist advice to classify as DM or not.

APDC = Admitted Patient Data Collection; NSWCR = New South Wales Cancer Registry; MBS = Medicare Benefits Schedule; PBS = Pharmaceutical Benefits Scheme; RBDM = Registry of Births, Deaths and Marriages

Validation of distant metastasis flags

We assessed the number of people with DM identified with each criterion/data source and reported the number of people with DM identified from multiple criterion/data sources in a Euler diagram (Figure 1). We considered the main risk of bias using the metastatic-specific criteria (1-4) alone to be the potential for delayed or incomplete ascertainment of the date of first DM because some people may initially be managed outside of hospital, using treatments not restricted to metastatic cancer, and without a pathology or radiation service to trigger a cancer registry notification. We developed metastatic pattern criteria 5 and 6 to help address this limitation. We considered the main risk of bias using these latter criteria as the potential for over-estimation of DM events. Thus, for people meeting criteria 5 or 6 as the *only* record of DM (ie. criteria 1-4 were not met in the study period); or occurring more than 6 months prior to criteria 1-4, we individually inspected their records from the NSWCR, APDC, PBS and MBS referring to within 90 days of the criterion date to exclude a locoregional recurrence, second primary BC or non-breast primary cancer before accepting the criterion as a DM event.

To estimate the number of DM events that may be missed using these methods, we assessed the number of deaths with BC listed as a primary or contributing cause of death but the person had not met the study's DM criteria.

Sensitivity analyses

We performed three sensitivity analyses to assess the impact of using more or less stringent criteria for recording DM on estimates of 5- and 10-year cumulative incidence: More stringent: (1) date of first DM estimated from metastatic-specific records only (criteria 1-4); Less stringent: (2) include date of BC death as a proxy for first date of DM for people who did not otherwise meet the DM criteria and for whom a treatment adverse event was not recorded as a contributing cause (ICD-10-AM Y43, 43.0-43.3); (3) accept the first date on which criteria 5 or 6 was met as the first date of DM, including those with concurrent records indicating locoregional recurrence or a second primary cancer.

Figure 1. Data sources for distant metastasis records

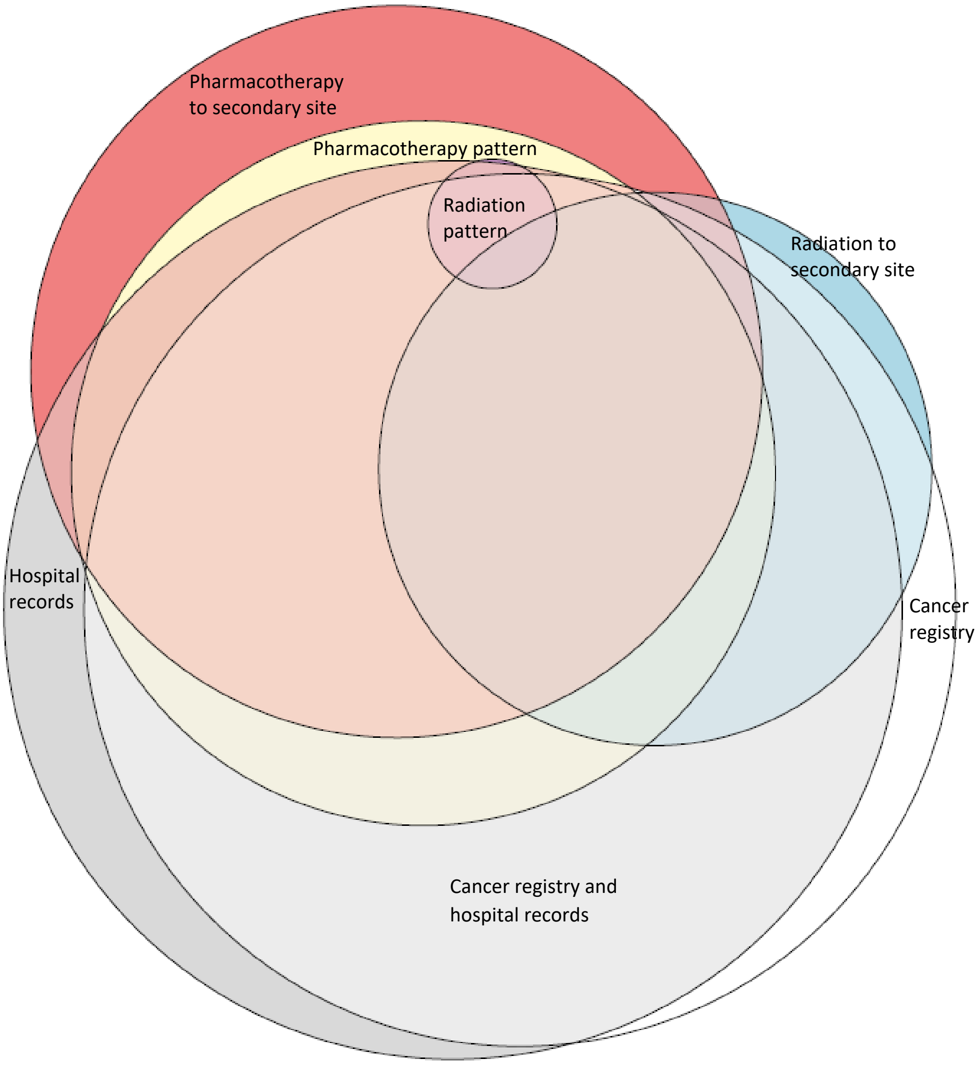

DM criteria	Number[1]	Source for earliest date[2]	Sole source[3]
**Metastatic-specific**			
Cancer registry	1027 (72%)	689 (48%)	46 (3%)
Hospital records	1113 (78%)	227 (16%)	77 (5%)
Radiation therapy (MBS)	446 (31%)	115 (8%)	19 (1%)
Pharmacotherapy (PBS)	770 (54%)	277 (19%)	124 (9%)
**Metastatic pattern**			
Pharmacotherapy (PBS)	718 (50%)	117 (8%)	24 (2%)
Radiation therapy (MBS)	39 (3%)		
**Total**		1425	290 (20%)

1. DM was identified from death records without meeting these DM criteria for additional 7 people.
2. The cancer registry is listed as the source for the earliest date for DM if recorded in the registry earlier or on the same date as other data sources.
3. For 167 (12%) people, DM was only identified from Pharmaceutical Benefits Scheme (PBS) pharmaceutical and/or Medicare Benefits Schedule (MBS) radiation items, with no cancer registry or hospital DM records in the study period.

Figure 2. Selection of cases for inclusion in our analysis

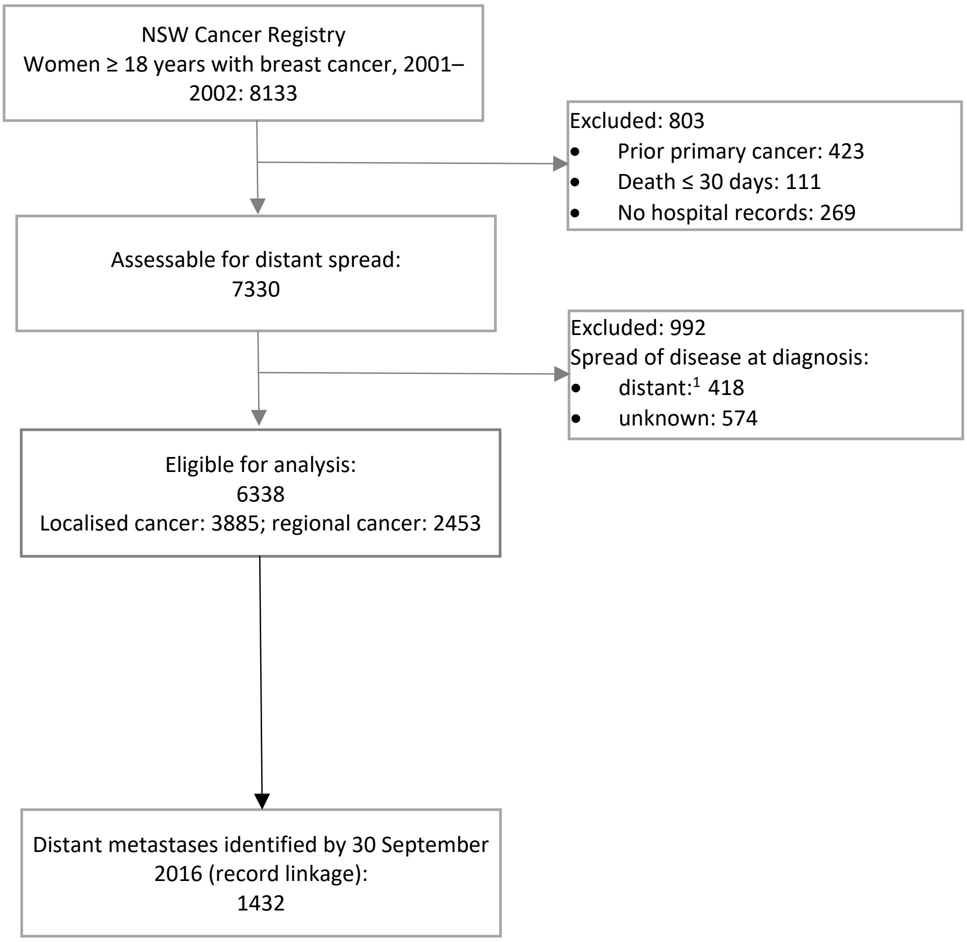

1. Includes distant metastasis recorded within 120 days of initial BC diagnosis

Table 2. Annual hazard rate for first distant metastasis by year since breast cancer diagnosis, by extent of disease at diagnosis

**Year[1]**	**0**	**1**	**2**	**3**	**4**	**5**	**6**	**7**	**8**	**9**	**10**	**11**	**12**	**13**	**Total**
**Localised** (T1-3 N0)															
n at risk	3885	3803	3647	3534	3450	3372	3290	3194	3106	3020	2949	2851	2766	2691	
n failed	60	104	66	39	33	29	38	29	31	21	25	26	26	24	570
n censored	22	52	47	45	45	53	58	59	55	50	73	59	49	429	3315
Annual hazard rate % (95% CI)	1.56 (1.17-1.96)	2.79 (2.26-3.33)	1.84 (1.39-2.28)	1.12 (0.77-1.47)	0.97 (0.64-1.30)	0.87 (0.55-1.19)	1.17 (0.80-1.54)	0.92 (0.59-1.26)	1.01 (0.66-1.37)	0.70 (0.40-1.00)	0.86 (0.52-1.20)	0.93 (0.57-1.28)	0.95 (0.59-1.32)	0.97 (0.58-1.36)	
**Regional** (T4 or N+)															
n at risk	2453	2328	2101	1952	1849	1765	1691	1628	1569	1513	1457	1403	1354	1312	
n failed	100	201	124	82	60	50	36	33	30	36	23	23	22	24	862
n censored	25	26	25	21	24	24	27	26	26	20	31	26	20	200	1591
Annual hazard rate % (95% CI)	4.18 (3.36-5.00)	9.08 (7.82-10.33)	6.12 (5.04-7.20)	4.31 (3.38-5.25)	3.32 (2.48-4.16)	2.89 (2.09-3.70)	2.17 (1.46-2.88)	2.06 (1.36-2.77)	1.95 (1.25-2.64)	2.42 (1.63-3.22)	1.61 (0.95-2.27)	1.67 (0.99-2.35)	1.65 (0.96-2.34)	2.00 (1.20-2.80)	

CI = confidence interval; DM = distant metastasis.

1. Refers to the beginning of each interval with the annual hazard rate calculated for mid-point of the interval e.g., for those alive and DM-free at the beginning of year 2 (24 months after a breast cancer diagnosis), the hazard rate of DM within the next 12 months (third year) is calculated at 2.5 years.

Figure 3. Annual hazard rate of distant metastasis, by time since breast cancer diagnosis and disease extent at diagnosis*

A. Age

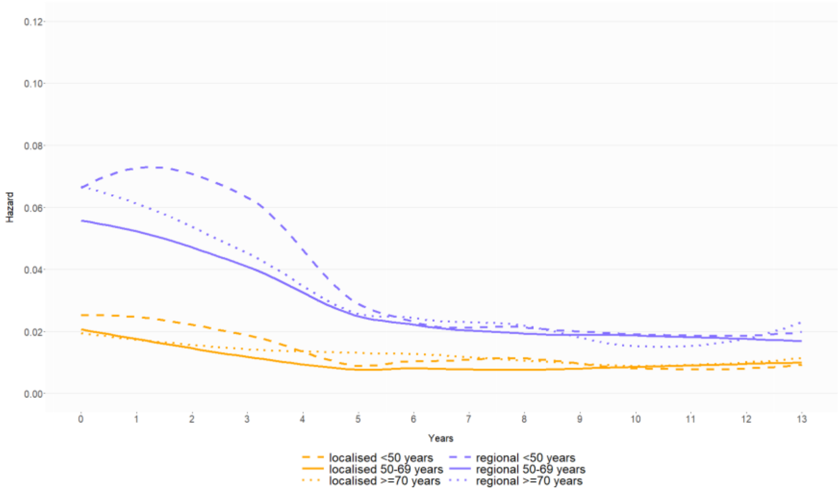

B. Tumour morphology

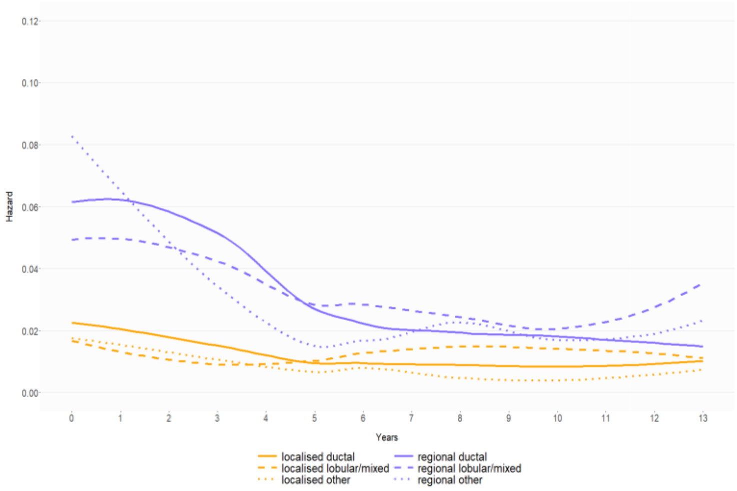

C. Treatment-defined ER-status

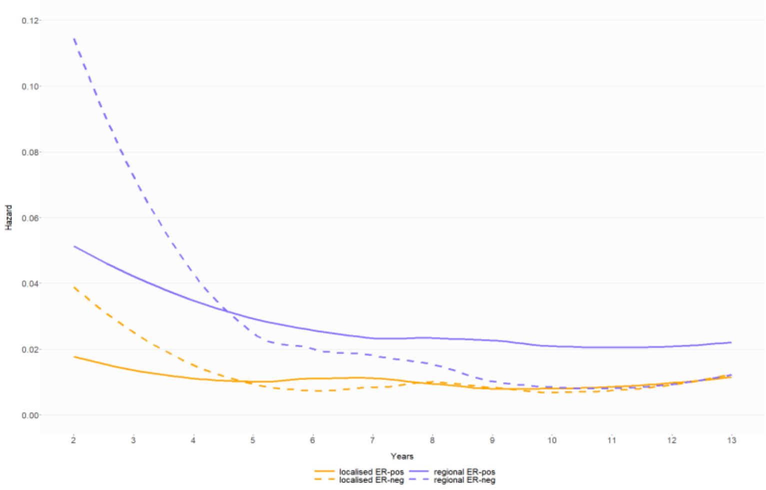

* The annual hazard rate of DM estimates the probability, for women alive and DM-free at the beginning of the interval, of experiencing DM during the subsequent 12 months .

Table 3. Annual hazard rate for breast cancer death and all-cause death, by year since first distant metastasis record

**Year**[1]	0	1	2	3	4	5	6	Total events to 30 June 2017
**All cause death**								
n at risk	1425	913	658	494	398	333	296	
n failed	505	220	129	70	44	22	15	1049
n censored	<10	35	35	26	21	15	21	376
Annual hazard rate % (95% CI)	43 (40-47)	28 (24-32)	22 (19-26)	16 (12-19)	12 (8-16)	7 (4-10)	5 (3-8)	
**Breast cancer death**								
n at risk	1425	913	658	494	398	333	296	
n failed	426	198	121	64	34	21	11	900
n censored	86	57	43	32	31	16	25	525
Annual hazard rate % (95% CI)	36 (33-40)	25 (22-29)	21 (17-25)	14 (11-18)	9 (6-12)	7 (4-10)	4 (2-6)	

CI = confidence interval.

1. Refers to the beginning of each interval with the annual hazard rate calculated in mid-point of the interval. e.g., for those alive at the beginning of year 3 (36 months after the first distant metastasis record), the annual hazard rate of death within the next 12 months (fourth year) is calculated at 3.5 years.

Figure 4. Annual hazard rate for breast cancer death during six years after first distant metastasis record

A. By extent of disease at breast cancer diagnosis

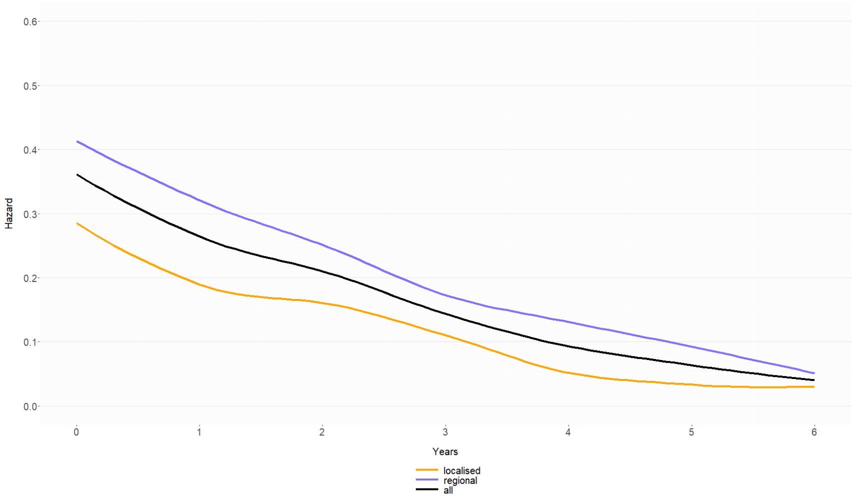

B. By age at first distant metastasis

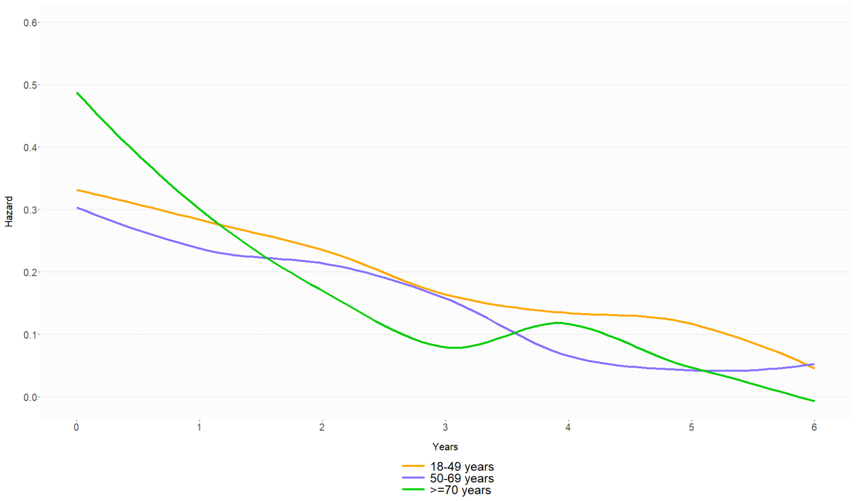

C. By tumour morphology

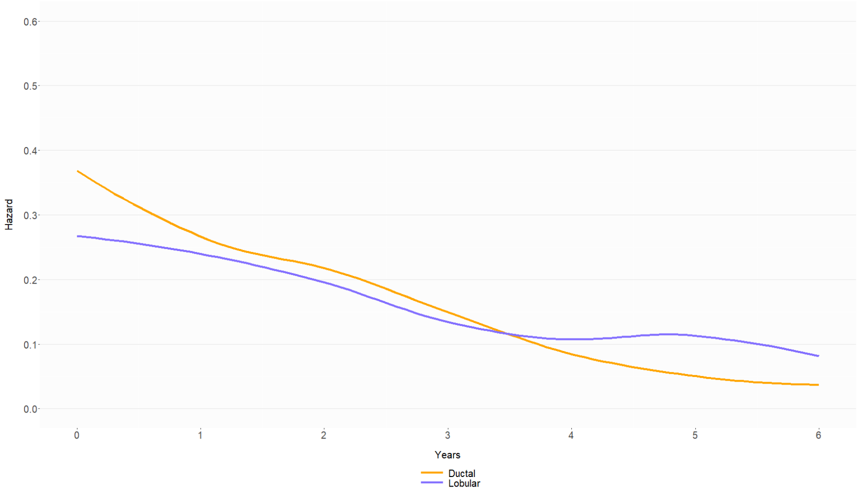

D. By distant metastasis-free interval

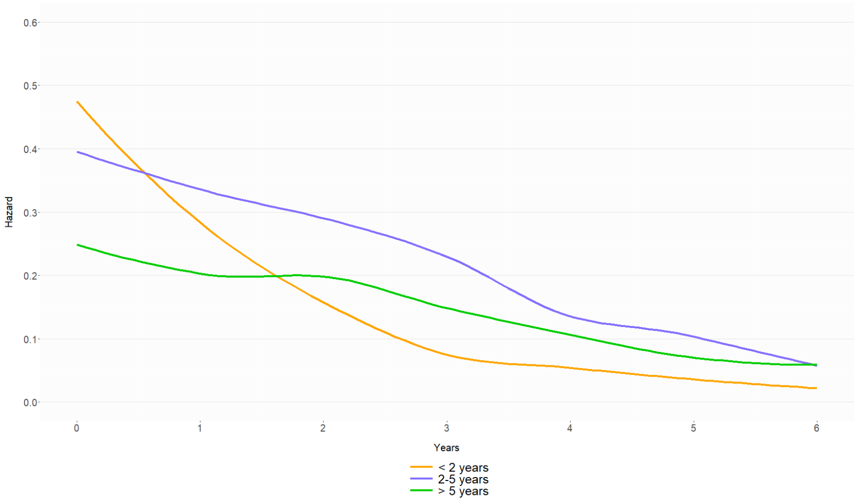

E. By treatment defined estrogen receptor status

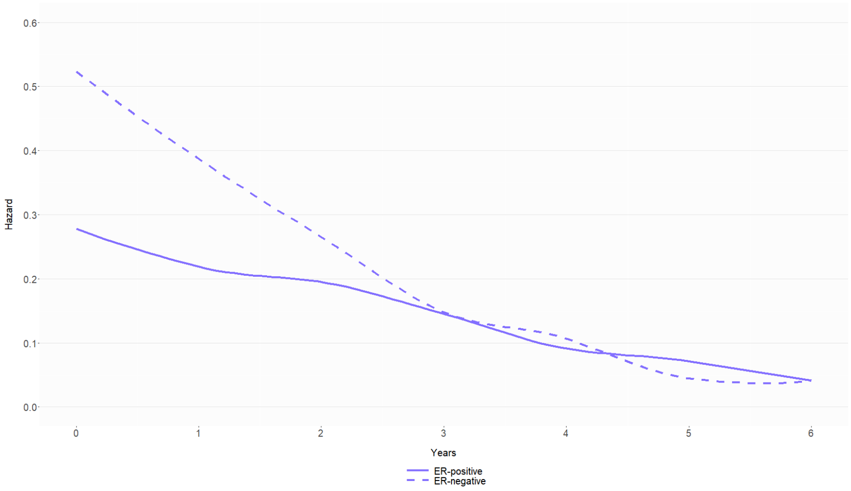

F. By visceral disease spread

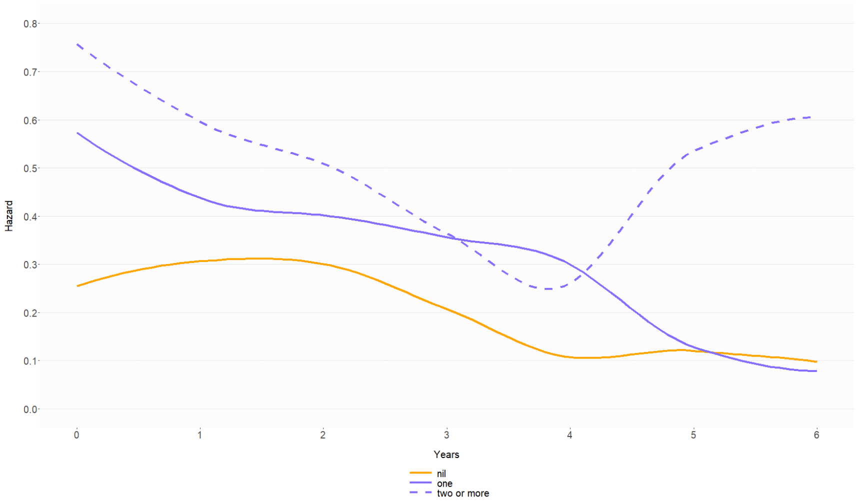

The annual hazard rate of BC death estimates the probability of BC death in a 1-year interval for individuals remaining alive at the beginning of the interval.

Table 4. Sensitivity analyses for estimation of: A. date of first distant metastasis (DM); and B. post-metastasis breast cancer-specific survival (BCSS)

A. Cumulative incidence of DM at breast cancer diagnosis

	Cumulative incidence % DM (95% CI)			
	localised		Regional	
**DM, N**	**5-year**	**10-year**	**5-year**	**10-year**
**Sensitivity analysis 1**				
DM criteria restricted to date of first metastatic-specific record (criteria # 1-4)				
1408	7.7 (6.9– 8.6)	11.4 (10.4– 12.4)	22.7 (21.1– 24.4)	30.3 (28.5– 32.2)
**Sensitivity analysis 2**				
DM criteria expanded to accept date of first metastatic treatment pattern (criteria 5 & 6) for people with records indicating concurrent locoregional recurrence or a second primary cancer				
1499	8.2 (7.3-9.0)	12.4 (11.3– 13.4)	23.6 (21.9– 25.3)	31.4 (29.6– 33.3)
**Sensitivity analysis 3**				
DM criteria expanded to include date of BC death if no DM recorded in death record or earlier (58 BC deaths reclassified as DM)				
1490	8.3 (7.5– 9.2)	12.3 (11.3– 13.4)	23.8 (22.1– 25.5)	31.6 (29.7– 33.4)

B. BCSS following DM

DM, N [1]	Median BCSS (IQR), months
**Sensitivity analysis 1**	
BCSS from date of first metastatic-specific record (criteria # 1-4)	
N=1401	27 (8 – not reached)
**Sensitivity analysis 2**	
BCSS from date of first DM criteria # 1-6, including first metastatic treatment pattern criteria 5 & 6 for people with records indicating concurrent locoregional recurrence or a second primary cancer	
N=1492	25 (6 – 127)

1. Excludes individuals with death as first DM record.

References

1. Australian Institute of Health and Welfare. BreastScreen Australia monitoring report 2021 (Cat. no. CAN 140). Canberra: AIHW, 2021. https://www.aihw.gov.au/reports/cancer-screening/breastscreen-australia-monitoring-report-2021/summary (viewed May 2022).

2. Australian Institute of Health and Welfare. BreastScreen Australia monitoring report 2000–2001 (Cat. no. CAN 20; Cancer Series no. 25). Canberra: AIHW, 2003. https://www.aihw.gov.au/reports/cancer/breastscreen-australia-monitoring-report-2000-2001/contents/table-of-contents (viewed May 2022).

3. Giuliano AE, Connolly JL, Edge SB, et al. Breast cancer: major changes in the American Joint Committee on Cancer eighth edition cancer staging manual. *CA Cancer J Clin* 2017; **67**: 290-303.
